# The *Symbiodinium* Proteome Response to Thermal and Nutrient Stresses

**DOI:** 10.1093/pcp/pcac175

**Published:** 2022-12-24

**Authors:** Clinton A Oakley, Grace I Newson, Lifeng Peng, Simon K Davy

**Affiliations:** School of Biological Sciences, Victoria University of Wellington, PO Box 600, Wellington 6140, New Zealand; School of Biological Sciences, Victoria University of Wellington, PO Box 600, Wellington 6140, New Zealand; School of Biological Sciences, Victoria University of Wellington, PO Box 600, Wellington 6140, New Zealand; School of Biological Sciences, Victoria University of Wellington, PO Box 600, Wellington 6140, New Zealand

**Keywords:** Coral bleaching, Eutrophication, Phosphorus limitation, Proteomics, *Symbiodinium*, Thermal stress

## Abstract

Coral bleaching is primarily caused by high sea surface temperatures, and nutrient enrichment of reefs is associated with lower resilience to thermal stress and ecological degradation. Excess inorganic nitrogen relative to phosphate has been proposed to sensitize corals to thermal bleaching. We assessed the physiological and proteomic responses of cultures of the dinoflagellate coral symbiont *Symbiodinium microadriaticum* to elevated temperature under low-nutrient, high-nutrient and phosphate-limited conditions. Elevated temperature induced reductions of many chloroplast proteins, particularly the light-harvesting complexes, and simultaneously increased the abundance of many chaperone proteins. Proteomes were similar when the N:P ratio was near the Redfield ratio, regardless of absolute N and P concentrations, but were strongly affected by phosphate limitation. Very high N:P inhibited *Symbiodinium* cell division while increasing the abundance of chloroplast proteins. The proteome response to phosphate limitation was greater than that to elevated temperature, as measured by the number of differentially abundant proteins. Increased physiological sensitivity to high temperatures under high nutrients or imbalanced N:P ratios was not apparent; however, oxidative stress response proteins were enriched among proteins responding to thermal stress under imbalanced N:P ratios. These data provide a detailed catalog of the effects of high temperatures and nutrients on a coral symbiont proteome.

## Introduction

Coral reefs are under threat from a combination of global stresses of climate change and ocean acidification as well as local stresses, such as overfishing and eutrophication (Hughes et al. 2018, Guan et al. 2020). Coral reefs are dependent on an endocellular mutualism between scleractinian corals and dinoflagellates of the family Symbiodiniaceae (LaJeunesse et al. 2018). These endosymbiotic algae exchange photosynthates for host-derived nitrogenous compounds and inorganic carbon (Davy et al. 2012, Rosset et al. 2021). Environmental perturbations can result in coral bleaching, a state where symbiont populations are reduced as a result of algal and host cellular stress responses (Oakley and Davy 2018). The principal cause of coral bleaching is elevated temperature, often combined with high irradiance, which can disrupt the dinoflagellate’s photosynthetic apparatus (Warner et al. 1999, Hill et al. 2011), leading to the formation of reactive oxygen species (ROS) and reactive nitrogen species (Hawkins and Davy 2013, Warner and Suggett 2016). It has been proposed that these reactive species may, in combination with those generated by host cells, elicit an immune response in the coral host, resulting in the removal of symbionts (Weis 2008, Saragosti et al. 2010, Tchernov et al. 2011, Pinzón et al. 2015, Rehman et al. 2016, Nielsen et al. 2018). If the symbiosis is not re-established, these ‘bleached’ corals suffer energy deprivation, increased susceptibility to disease and mortality (McClanahan et al. 2019, Vargas-Ángel et al. 2019).

While coral bleaching is primarily caused by increased sea surface temperatures resulting from carbon emissions (Hughes et al. 2017), the importance of the local nutrient environment in determining the susceptibility and resilience of corals to thermally induced bleaching is becoming increasingly apparent (D’Angelo and Wiedenmann 2014, Lapointe et al. 2019, Guan et al. 2020). Nitrogen and phosphorus that enter coastal systems via eolian dust and riverine, diffuse and engineered discharge sources, and human populations along coastlines dramatically influence coastal ecosystems through nutrient inputs. Coral reefs, which typically thrive in oligotrophic waters, are particularly vulnerable to eutrophication (Porter and Muscatine 1977, Morris et al. 2019). Worldwide, increased nutrient loading has been linked to the degradation of coral reefs in regions where they are in close proximity to urbanization, agriculture or industry (Fabricius 2005, Wooldridge 2009, Lapointe et al. 2019).

Nitrogen-enriched runoff due to agricultural reliance on synthetic nitrogen fertilizers or nutrient disruptions from phytoplankton blooms has been suggested as a mechanism by which the nitrogen:phosphorus (N:P) ratio of the reef environment may exceed the 16:1 Redfield ratio (D’Angelo and Wiedenmann 2014, Lapointe et al. 2019). Under nitrogen-enriched conditions, Symbiodiniaceae growth rates increase (Fabricius 2005, Cunning and Baker 2013, Rosset et al. 2015), increasing the demand for carbon and phosphate. Imbalances in the relative availability of nitrogen and phosphorus have been shown to greatly affect Symbiodiniaceae physiology, imperiling the symbiosis (Wiedenmann et al. 2013, Rosset et al. 2017, Fernandes de Barros Marangoni et al. 2020). The ratio of N:P in particulate organic matter is somewhat variable between ocean basins and phytoplankton groups, but dinoflagellates in culture can exhibit a cellular N:P near that of the 16:1 Redfield ratio (Sharoni and Halevy 2020). Phosphate starvation of Symbiodiniaceae has been shown to induce a shift in the lipid composition of the thylakoid membrane from phospholipids to sulfolipids, which is thought to reduce the threshold for thermal- and light-induced damage by decreasing photosystem integrity (Wiedenmann et al. 2013). Recent studies have found that symbiont photosynthetic dysfunction, thermally induced bleaching and a reduced translocation of photosynthates by symbionts are greater under a severely imbalanced nutrient regime in favor of nitrogen (Ezzat et al. 2015, 2016, Rosset et al. 2017) and that elevated nitrogen (especially as nitrate) concentrations exacerbate thermal bleaching and ROS generation (Fernandes de Barros Marangoni et al. 2020). While the physiological and phenotypic responses of Symbiodiniaceae to elevated nutrients and temperature are well-characterized, the cellular mechanisms that underlie these responses are not well understood.

In order to provide a detailed, protein-level study of the independent and combined effects of thermal and nutrient stressors on dinoflagellate coral symbionts, we performed label-free quantitative proteomics using liquid chromatography-tandem mass spectrometry (LC–MS/MS). In combination with physiological measurements, analysis of the proteome provides high-resolution details of the cellular phenotype and has not been performed previously on intact Symbiodiniaceae (Tortorelli et al. 2022). Thermal stress and oxidative stressors not only change protein transcription but may also directly disrupt protein synthesis, folding, repair and degradation rates, and so proteomic assessments may be better able to capture stress effects than transcript-based studies (Vogel et al. 2011). Using a full factorial design, *Symbiodinium microadriaticum* cultures were grown at 25 and 34°C under low- (no additional N), high- (N and P added) or imbalanced (only N added) nutrient conditions. N and P concentrations and ratios were chosen to represent ecologically relevant coral reef scenarios. Cell population growth, photosynthetic rate and efficiency, respiration rate and phosphatase activity were assessed to corroborate trends of protein expression. Algal proteins were quantified, and gene ontology (GO) terms were used to conduct enrichment analysis on cellular processes. We hypothesized that protein expression would change in response to both temperature and nutrient regimes, particularly photosynthetic proteins and those involved in oxidative stress, and that there would be an interaction between these two environmental factors on the dinoflagellate proteome. We also predicted that cultures exposed to an imbalanced nutrient regime would show greater susceptibility to thermal stress, as observed in other studies (Wiedenmann et al. 2013, Rosset et al. 2017).

## Results and Discussion

### Physiological measurements

**Fig. 1:**
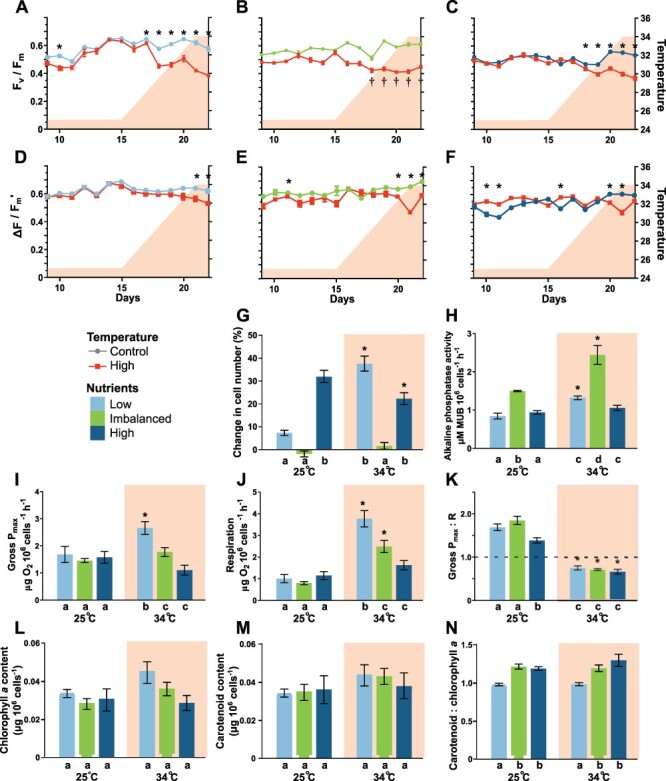
Physiological parameters of *S. microadriaticum* cultures under different thermal and nutrient regimes. (A–C) The maximum quantum efficiency of photosystem II (*F*_v_/*F*_m_) of *S. microadriaticum* cultures under low-, imbalanced and high-nutrient regimes (*n* = 8). (D–F) The effective quantum efficiency of photosystem II (Δ*F*/*F*_m_′). Shading indicates the temperature of the elevated temperature treatment. (G) The difference in cell abundance (counts) in each culture vessel from the beginning to the end of the experimental period (*n* = 8–10). (H) Alkaline phosphatase activity (*n* = 4). (I) Maximum gross photosynthetic rate. (J) Respiratory oxygen consumption. (K) The ratio of maximum gross photosynthesis to respiration (*n* = 3–4). (L–K) Chlorophyll *a*, carotenoid and carotenoid:chlorophyll *a* content. The values are mean ± standard error. ‘Low’, ‘imbalanced’ and ‘high’ represent different nutrient regimes. * indicates significant differences between the 25 and 34°C treatments as determined by *t*-tests, and † indicates significant differences over time from the beginning of the experiment by repeated measures ANOVA. Letters indicate significant differences as determined by a Kruskal–Wallis test across all treatments (G) or two-way ANOVA followed by a simple main effects analysis within each temperature treatment (H–N). MUB, methylum belliferyl.

The physiological effects of high temperature were broadly similar across nutrient conditions (**Fig. 1**). High temperatures resulted in reduced quantum efficiency of photosystem II (PSII) under all nutrient conditions (**Fig. 1A****–****C**), as well as significant but minor reductions in the effective quantum efficiency of PSII (**Fig. 1D****–****F**). High temperature under low-nutrient conditions resulted in an increased cell population, alkaline phosphatase activity, maximum gross photosynthetic rate and respiration rate (**Fig. 1G****–****J**). Cell populations, reflecting division rates, were greater under high-nutrient conditions than under low-nutrient conditions at 25°C but were similar at 34°C. Notably, there was no increase in the algal cell population under the imbalanced nutrient regime regardless of temperature. Alkaline phosphatase activity increased with temperature under both low- and imbalanced nutrient regimes (**Fig. 1H**). Regardless of temperature, alkaline phosphatase activity was highest under the imbalanced regime. The gross maximum photosynthetic rate increased with temperature under low-nutrient conditions but not under imbalanced or high-nutrient conditions (**Fig. 1I**). Respiration increased with temperature under low- and imbalanced nutrient regimes (**Fig. 1J**). Gross P_max_:R was below compensation (i.e. <1) at 34°C under all nutrient conditions (**Fig. 1K**). Chlorophyll *a* and carotenoid content were unaffected by temperature (**Fig. 1L****–****N**).

### Proteome results

**Fig. 2:**
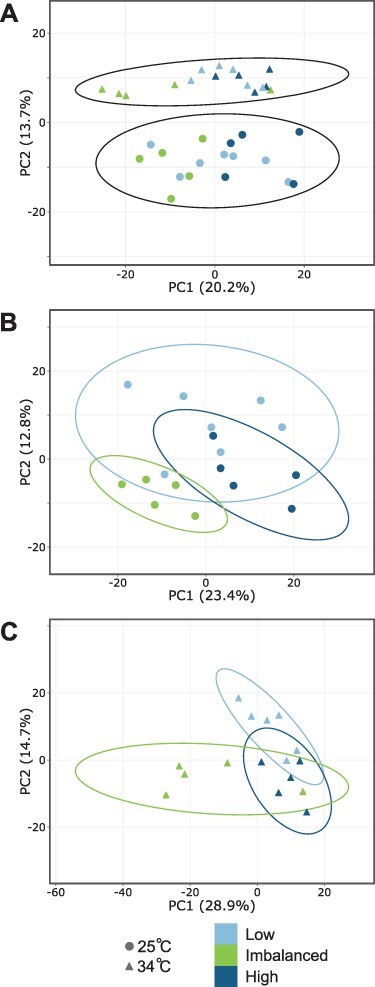
Principal component analysis of label-free quantification intensity values of *S. microadriaticum* protein groups under different thermal and nutrient regimes. (A) All samples grouped by thermal treatment. (B) Samples at 25°C, grouped by the nutrient regime. (C) Samples at 34°C, grouped by the nutrient regime. Each point represents one biological replicate. Unit variance scaling is applied to rows, and singular value decomposition with imputation is used to calculate principal components. Ellipses indicate 95% confidence intervals for each treatment.

**Fig. 3:**
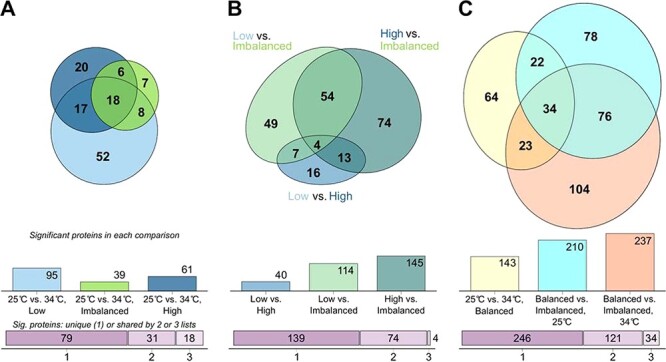
The effects of temperature and low-, imbalanced and high-nutrient regimes on the proteome and protein functions of *S. microadriaticum*. (A) Significantly DAPs between 25 and 34°C at low-, imbalanced and high-nutrient regimes. (B) Differentially abundant proteins between each nutrient condition. (C) Differentially abundant proteins between *S. microadriaticum* cells at 25 and 34°C under balanced (low + high nutrient samples), balanced compared with imbalanced N:P at 25°C and balanced compared with imbalanced N:P at 34°C. The areas of each ellipse are proportional to their contents. Bar charts provided for direct comparison of the size of each comparison. Full lists of each protein set are available in **Supplementary Materials S1, S3–S5**.

A total of 1,292 proteins were identified by searches against *S. microadriaticum* sequences. Principal component analysis of protein quantities demonstrated clear separation of the two temperature treatments (**Fig. 2A**). At 25°C, the results obtained low- and high-nutrient conditions largely overlapped, while the imbalanced nutrient condition showed some separation(**Fig. 2B**). This separation was more distinct at 34°C (**Fig. 2C**). A total of 128 differentially abundant proteins (DAPs) were detected when comparing 25°C vs. 34°C in separate nutrient treatments (**Table 1** and **Fig. 3A**; **Supplementary Material S1**). When all data were analyzed simultaneously, 161 proteins responded to temperature (‘thermal DAPs’; **Table 1**). Within individual nutrient treatments, there were 95, 39 and 61 thermal DAPs at low-, imbalanced and high-nutrient conditions, respectively (**Table 1** and **Fig. 3A**). While many of these were shared between two or more nutrient conditions (49 of 128 = 38%; **Fig. 3A**), the low nutrient treatment had the largest number of unique thermal DAPs (52 of 79 = 66%). Moreover, not only 18 thermal DAPs were shared under all nutrient conditions, primarily chaperonins and heat shock proteins, but also Rubisco, the photosystem II D2 protein and apocytochrome f (**Fig. 3A**; **Supplementary Material S1**). Gene ontology biological process terms related to photosynthesis, protein folding, lipid catabolism and glycolysis were significantly enriched with DAPs (**Table 2**; **Supplementary Material S2**). We next compared the effects of nutrient availability on the proteome. When compared against the imbalanced treatment, the low- and high-nutrient treatments had 114 and 145 DAPs, respectively, 58 of which were shared (**Fig. 3B**). Algal proteomes under low and high nutrients were more similar, exhibiting only 40 DAPs between them (**Fig. 3B**).

**Table 1:** Significantly differentially abundant proteins (FDR <5%) between thermal and nutrient regimes

	Differentially abundant proteins (*n*)	
Nutrient treatments		
Low vs. high	40 (13, 10)**^a^**	
Low vs. imbalanced	114 (13, 10)	
High vs. imbalanced	145 (10, 10)	
Balanced vs. imbalanced	322 (23, 10)	
25°C vs. 34°C		
All treatments	161 (17, 16)	
Low	95 (6,7)	
Imbalanced	39 (5,5)	
High	61 (5,5)	
Balanced (low and high)	143 (12, 11)	
Combined treatments	*Temperature treatment*	
	25°C	34°C
Balanced vs. imbalanced	210 (12, 5)	237 (11, 5)

a Numbers in brackets indicate the number of samples in each of the compared treatments.

**Table 2:** GOterms that were enriched (*P* < 0.1) among DAPs when comparing 25°C vs. 34°C under balanced nutrient conditions. Full results are available in **Supplementary Materials S2**

25°C vs. 34°C and Balanced N:P			
GO ID	GO term	DAPs	*P*
Biological process			
GO:0009772	Photosynthetic electron transport in photosystem II	3	0.004
GO:0015979	Photosynthesis	18	0.0043
GO:0006457	Protein folding	8	0.0072
GO:0018298	Protein–chromophore linkage	6	0.0142
GO:0044242	Cellular lipid catabolic process	2	0.0256
GO:0006096	Glycolytic process	3	0.0863
Cellular compartment			
GO:0009535	Chloroplast thylakoid membrane	2	0.02
GO:0005783	Endoplasmic reticulum	3	0.022
GO:0009507	Chloroplast	5	0.091
GO:0009523	Photosystem II	2	0.099
GO:0005886	Plasma membrane	2	0.099
Molecular function			
GO:0016810	Hydrolase activity, acting on carbon–nitrogen (but not peptide) bonds	2	0.022
GO:0045156	Transferring electrons within the cyclic electron transport pathway of photosynthesis activity	2	0.022
GO:0016168	Chlorophyll binding	12	0.026
GO:0005524	ATP binding	21	0.049
GO:0102250	Linear malto-oligosaccharide phosphorylase activity	2	0.058
GO:0008184	Glycogen phosphorylase activity	2	0.058
GO:0046872	Metal ion binding	20	0.073

**Fig. 4:**
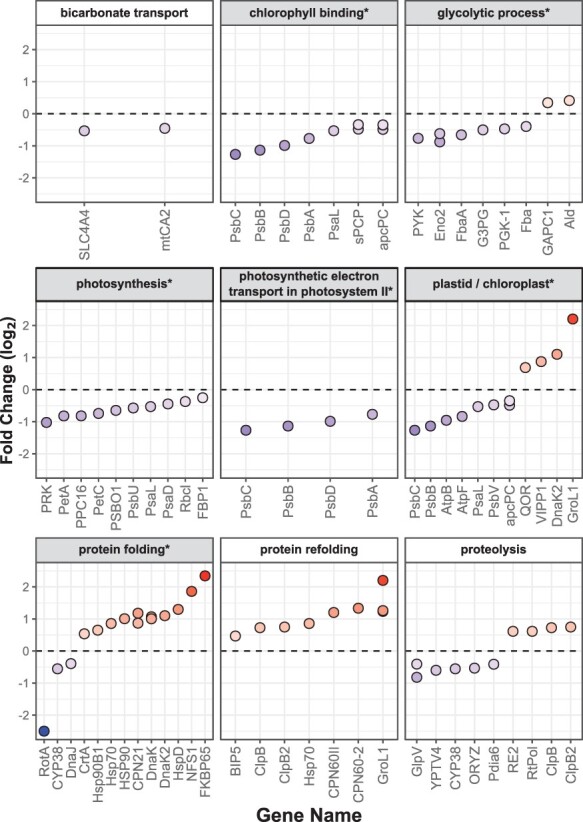
*Symbiodinium microadriaticum* proteins that were differentially abundant between 25 and 34°C under balanced nutrient conditions, grouped by selected GO terms. Gene ontology terms that were enriched in DAPs using topGO (FDR < 0.1, **Table 2**) are marked by shading with an asterisk. Proteins are sorted by log_2_ FC, where positive values indicate proteins, which were more abundant at 34°C. Proteins whose negative FC was infinite are displayed with a value of −2.5. Uncharacterized proteins and those appearing in multiple plots have been removed for clarity.

**Fig. 5:**
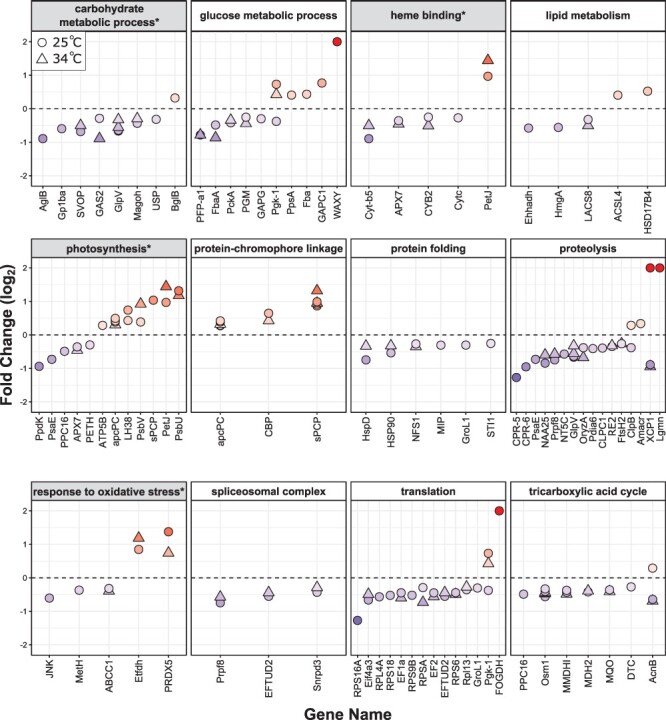
Selected GO terms enriched in *S. microadriaticum* proteins that were differentially abundant between balanced (low + high) and imbalanced nutrient conditions. Gene ontology terms that were enriched in DAPs using topGO (FDR < 0.1) are marked by shading with an asterisk. Proteins are sorted by log_2_ FC, where positive values indicate proteins that were more abundant under imbalanced N:P conditions. The temperature is indicated by the symbol shape. Proteins that were only significant at one temperature are included, and those whose positive fold-change was infinite are displayed with a value of 2. Uncharacterized proteins and those appearing in multiple plots have been removed for clarity.

To directly compare the relative effect sizes of thermal stress and imbalanced N:P ratios on the *S. microadriaticum* proteome (as measured by the number of DAPs), the low and high nutrient samples were combined into a single ‘balanced’ condition for further analysis. This pooling was supported by the overlapping distributions of samples by principal components (**Fig. 2A****–****C**) and the small number of DAPs when comparing low- vs. high-nutrient conditions (**Fig. 3B**). Among all balanced nutrient samples (composed of all high- and low-nutrient samples), exposure to 34°C resulted in 143 thermal DAPs (**Table 1**, **Fig. 3C**; **Supplementary Material S3**). This was a smaller effect than comparing the balanced vs. imbalanced nutrient conditions (‘nutrient DAPs’) at either temperature (210 nutrient DAPs at 25°C and 237 nutrient DAPs at 34°C; **Fig. 3C**, **Supplementary Material S4** and **S5**). Comparisons between the balanced and imbalanced nutrient conditions were made at both 25 and 34°C to determine if there were additive effects of combining high temperature with imbalanced nutrient availability. Many of the thermal DAPs in the balanced nutrient treatment (143) were a subset of the nutrient DAPs at either temperature (**Fig. 3C**). The nutrient DAPs at 25 or 34°C also strongly overlapped (**Fig. 3C**). The large proteomic effects of an imbalanced N:P ratio, despite its limited apparent physiological effects on photosynthesis, indicate both acclimation and stress on many of the same cellular processes affected by high temperature (**Figs. 3C**, **4**, **5**). Gene ontology biological process terms related to photosynthesis, carbohydrate and lipid metabolism were enriched with nutrient DAPs at 25°C (**Table 3**), and those related to photosynthesis, oxidative stress, and lipid metabolism were enriched with nutrient DAPs at 34°C (**Table 4**; **Supplementary Material S2**).

**Table 3:** GO terms that were enriched (*P* < 0.1) among DAPs when comparing balanced vs. imbalanced N:P conditions at 25°C. Full results are available in **Supplementary Materials S2**

Balanced vs. imbalanced N:P, 25°C			
GO ID	GO term	DAPs	*P*
Biological process			
GO:0009765	Photosynthesis, light harvesting	17	0.0065
GO:0005975	Carbohydrate metabolic process	22	0.0267
GO:0006094	Gluconeogenesis	4	0.0823
GO:0045454	Cell redox homeostasis	2	0.0995
GO:0044242	Cellular lipid catabolic process	2	0.0995
GO:0009423	Chorismate biosynthetic process	2	0.0995
Cellular compartment			
GO:0016020	Membrane	76	0.0089
GO:0005783	Endoplasmic reticulum	4	0.0188
GO:0009507	Chloroplast	5	0.0574
GO:0030906	Retromer, cargo-selective complex	2	0.0698
GO:0009579	Thylakoid	4	0.0699
Molecular function			
GO:0003924	GTPase activity	6	0.0065
GO:0020037	Heme binding	12	0.0116
GO:0046933	Proton-transporting ATP synthase activity, rotational mechanism	4	0.0121
GO:0016747	Transferring acyl groups other than amino-acyl groups	5	0.0364
GO:0004721	Phosphoprotein phosphatase activity	5	0.0864

**Table 4:** GO terms that were enriched (*P* < 0.1) among DAPs when comparing balanced vs. imbalanced N:P conditions at 34°C. Full results are available in **Supplementary Materials S2**

Balanced vs. imbalanced N:P, 34°C			
GO ID	GO term	DAPs	*P*
Biological process			
GO:0015979	Photosynthesis	23	0.047
GO:0009765	Photosynthesis, light harvesting	14	0.065
GO:0006979	Response to oxidative stress	4	0.072
GO:0017001	Antibiotic catabolic process	2	0.092
GO:0044242	Cellular lipid catabolic process	2	0.092
GO:0009636	Response to toxic substance	2	0.092
GO:0006730	One-carbon metabolic process	2	0.092
Cellular compartment			
GO:0016020	Membrane	85	0.011
GO:0005783	Endoplasmic reticulum	4	0.023
GO:0009507	Chloroplast	4	0.068
GO:0019898	Extrinsic component of membrane	2	0.078
GO:0009579	Thylakoid	4	0.078
Molecular function			
GO:0020037	Heme binding	13	0.002
GO:0008484	Sulfuric ester hydrolase activity	4	0.01
GO:0005524	ATP binding	41	0.031
GO:0016747	Transferase activity, transferring acyl groups other than amino-acyl groups	5	0.032
GO:0016861	Intramolecular oxidoreductase activity, interconverting aldoses and ketoses	3	0.032
GO:0004601	Peroxidase activity	3	0.098
GO:0004190	Aspartic-type endopeptidase activity	3	0.098
GO:0046933	Proton-transporting ATP synthase activity, rotational mechanism	3	0.098
GO:0030170	Pyridoxal phosphate binding	3	0.098
GO:0004672	Protein kinase activity	6	0.099

Differential expression analysis of GO terms indicated that some of the same biological processes, principally photosynthesis and lipid metabolism, and cellular compartments, principally the chloroplast, thylakoid and endoplasmic reticulum, were most affected by both thermal and imbalanced nutrient stressors (**Tables 2**–**4**). The hypothesized amplification of, or sensitivity to, the thermal stress of *S. microadriaticum* under an imbalanced N:P ratio was not readily observed in the physiological responses or at the individual protein level (**Figs. 1**, **3**), in contrast to other studies (Wiedenmann et al. 2013, Rosset et al. 2017). Subtle combined effects of thermal and nutrient stress on the proteome could be detected, however, using differential expression analysis of GO terms. The effects of high temperatures and imbalanced nutrients are discussed further, both separately and in combination.

### Thermal effects on the *S. microadriaticum* proteome under balanced N:P

The effects of high temperature on the *S. microadriaticum* proteome in the absence of nutrient stress were significant, although smaller than those of an imbalanced N:P regime, as measured by the number of DAPs (**Table 1**, **Fig. 3C**). High temperature resulted in increases in proteins involved in protein folding and repair and decreases in those involved in both the light-harvesting and carbon-fixing processes of photosynthesis (**Fig. 4**). Differential expression analysis of GO terms indicated thermal effects on photosynthesis, photosynthetic electron transport in PSII, glycolysis, lipid catabolism and protein folding (**Table 2**). Given the limited physiological impairment, we therefore assume that the DAPs noted here represent those proteins greatly responsible for thermal acclimation in *Symbiodinium*.

Broadly, the phenotypic response to thermal stress was a reduction in chloroplast proteins, including those associated with both light-harvesting and carbon-fixation processes, while oxygen evolution rates were little changed. The photoinhibitory effects of thermal stress are well-studied in Symbiodiniaceae (Warner et al. 1999, Weis 2008, Warner and Suggett 2016), and here quantum efficiencies of PSII declined with temperature, consistent with moderate photoinhibition (Warner et al. 1996). Thermal stress induced a broad reduction in the abundance of many photosynthesis proteins, including soluble peridinin-chlorophyll *a* protein (sPCP) and chlorophyll *a*-chlorophyll *c*_2_-peridinin-protein (apcPC; appearing in UniProt as ‘fucoxanthin-chlorophyll a-c-binding protein A/C’, likely a mis-annotation; Jiang et al., 2014), components of the photosynthetic electron transport chain and both the photosystem I (PSI) and PSII reaction centers (**Fig. 4**). Both D1 and D2 proteins (PsbA and PsbD; Warner et al., 1999) and reaction center proteins PsbB and PsbC were all less abundant under thermal stress, in agreement with transcriptomic studies (Bellantuono et al. 2019). Proteins involved in the Calvin cycle were also largely diminished at 34°C (e.g. FbaA, PRK, FBP1, GAPC1 and PPC16), including form II ribulose bisphosphate carboxylase/oxygenase (RbcL). Rubisco activity has been found to be reduced at elevated temperatures (Lilley et al. 2010), including in the Symbiodiniaceae proteome (Mayfield et al. 2018). Inorganic carbon fixation and uptake proteins were also diminished, including carbonic anhydrase (mtCA2; the cellular compartment of this protein is undetermined), a bicarbonate transporter (SLC4A4) and multiple proton transporters (SCN5A, DUR3, NHAD and PMA-1; **Fig. 4**). Two components of cytochrome b_6_f, apocytochrome f (PETA) and the iron–sulfur subunit (Rieske protein, PETC), had lower abundance at 34°C. Cytochrome b_6_f abundance is central to regulating photosynthetic rates and cyclic electron flow around PSI, especially in response to environmental stress (McCabe Reynolds et al. 2008, Roberty et al. 2014, Schöttler et al. 2015), and so this finding is in contrast to the reported increase in cyclic electron flow around PSI at high temperatures in *Symbiodinium* (Dang et al. 2019).

This uniform reduction in chloroplast protein abundance at elevated temperature may be a consequence of non- or post-translational changes in protein abundance, including differing rates of repair, degradation and turnover (Leggat et al. 2011, Liu et al. 2016). It may also represent an acclimatory reduction in chloroplast volume in response to thermal stress while maintaining a similar photosynthetic rate and efficiency (**Fig. 1A****–****F, I**). Under low nutrients, the maximum photosynthetic rate increased at 34°C (**Fig. 1I**), despite significant reductions in chloroplast proteins, likely as a result of increased enzyme activity at elevated temperatures. Transcriptomic studies of Symbiodiniaceae under thermal stress have found variable expression of photosynthesis proteins (Barshis et al. 2014, Gierz et al. 2017). The relative contributions of transcriptional, translational and post-translational processes on protein abundance in the Symbiodiniaceae, as well as processes affecting protein lifetimes, deserve further study, particularly a comparison of steady state and stressed states.

The effects of thermal stress on the oxidation–reduction (redox) processes of the dinoflagellate symbiont have been an area of particular focus (Warner and Suggett 2016). Here, we detected abundance changes in several proteins involved in regulating mitochondrial and photosynthetic electron transport chains, and the GO process ‘photosynthetic electron transport in PSII’ was enriched among thermal DAPs (**Table 2** and **Fig. 4**). Chloroplast acclimation to shifts in electron flow is inferred from the broad decline in photosynthetic proteins as well as an increase in chloroplast quinone oxidoreductase, which is thought to detoxify peroxidized oxylipins spontaneously generated under oxidative conditions (Mas y mas et al. 2017). VIPP1, which promotes thylakoid membrane stability (Vothknecht et al. 2012), was more abundant at 34°C. In the mitochondria, a non-proton translocating electron transport chain enzyme, alternative nicotinamide adenine dinucleotide phosphate, reduced [NAD(P)H]-ubiquinone oxidoreductase B1 (NDB1), was less abundant under thermal stress. NDB1 serves to remove excess pressure on the mitochondrial electron transport chain by serving as an electron sink without producing adenosine triphosphate (ATP), and other non-proton translocating alternative oxidases have been shown to have increased activity under thermal stress (Oakley et al. 2014, Vega de Luna et al. 2020). We did not detect concerted increases in antioxidant mechanism proteins at 34°C, instead noting a surprising decrease in superoxide dismutase and flavodoxin abundances (SodA and FldA; **Fig. 4**). In total, and considering previous studies, we interpret these data as indicating cellular acclimation of electron transport chains to increased, but sublethal, stress.

The reduction in photosynthetic protein abundance may reflect the thermal down regulation of photosynthetic proteins in addition to the inhibition of photosystem repair via the de novo synthesis of proteins (Takahashi et al. 2004, 2008). Thermal stress greatly affected a suite of protein chaperones, heat shock proteins and other proteins involved in protein folding and repair, including HSP70, HSP90, HSP90B1, GroL1, DNAK, DNAK2, CPN60, CLPB and CLPB2 (**Fig. 4**). These proteins are localized in multiple locations throughout the cell, including the chloroplast, mitochondrion, endoplasmic reticulum and cytoplasm (**Supplementary Material S6**). Increases in DNAK2, CLPB, CLPB2 and two CPN21 proteins, all chaperones that are likely localized to the chloroplast by homology, likely reflect an increased need for protein stabilization and repair in the photosynthetic apparatus at high temperatures. Elevated temperatures are well-known to cause increased rates of protein misfolding and degradation in eukaryotes, as well as protein damage due to oxidative stress (Finke et al. 2015, Oakley et al. 2017), including in the transcriptomic analysis of Symbiodiniaceae (Rosin et al. 2011, Gierz et al. 2017). Proteolysis proteins were also affected by thermal stress here, including increases in CLPB, CLPB2 and RE2, as well as increases in a probable polyubiquitin (UBQ-1) (**Fig. 4**). Other proteins involved in proteolysis or protein folding (e.g. RotA, PDIA6 and CYP38) were less abundant under thermal stress, possibly reflecting the complexity of balancing protein folding, repair, stability and degradation.

### Nutrient effects on *S. microadriaticum*

The N:P ratio of the culture medium had a strong influence on the *S. microadriaticum* proteome (**Figs. 2**–**3**, **5** and **Tables 1**, **3**, **4**). There were relatively few differences between the algal proteomes under the low- vs. high-nutrient regime (40 DAPs; **Table 1** and **Fig. 3B**). However, when comparing these regimes with the imbalanced nutrient treatment, marked proteome differences were apparent (**Table 1** and **Figs. 2**, **3**). Physiological and proteomic data indicated that the algae under an imbalanced nutrient regime were experiencing P deficiency, as has been previously observed in Symbiodiniaceae at high levels of N relative to P (Wiedenmann et al. 2013, D’Angelo and Wiedenmann 2014, Rosset et al. 2017). Alkaline phosphatase activity increased under imbalanced N:P at 34°C compared to 25°C (**Fig. 1H**), likely reflecting even greater phosphate demand at high temperatures under phosphate limitation. Unfortunately, alkaline phosphatases, while present in the *S. microadriaticum* genome, were not detected by mass spectrometry and may therefore be present in relatively low abundance.

Given little differentiation between the responses to the low- and high-nutrient levels, and our focus on the effects of an imbalanced N:P ratio, for further analysis, the low- and high-nutrient treatments were combined into a single ‘balanced’ condition for comparison to the imbalanced condition at both 25 and 34°C. Approximately half of the nutrient DAPs at each temperature were shared, with many enriched GO terms shared between the two (**Fig. 3C** and **Tables 3**, **4**). Furthermore, when individual nutrient DAPs were assessed, their direction of change at 25 and 34°C was highly uniform, with few showing opposing positive/negative fold-changes (FCs) in response to temperature (**Fig. 5**). We will, therefore, discuss the effects of imbalanced N:P on the proteome independent of temperature, while calling attention to those proteins or processes where interacting effects were determined.

Several photosystem proteins exhibited increased abundances under the imbalanced N:P regime relative to the balanced state, including the highly abundant apcPC and sPCP proteins (**Fig. 5**). There was no substantial effect of nutrient availability on PSII quantum efficiency (**Fig. 1A****–****F**), in accordance with previous studies that have found it to be insensitive to nutrient availability in both non-symbiotic diatoms and Symbiodiniaceae *in hospite* (Parkhill et al. 2001, Wiedenmann et al. 2013). Gross photosynthetic rate was also unaffected by nutrient availability at 25°C (**Fig. 1I**). However, relative to the low- and high-nutrient regimes, *S. microadriaticum* under the imbalanced regime had severely depressed population growth, indicating phosphorus limitation (**Fig. 1G**). Three Calvin cycle proteins, PGK-1, GAPC1 and FBA, were more abundant under the imbalanced regime (carbohydrate metabolic process; **Fig. 5**), while Rubisco itself was unaffected. Two distinct glyceraldehyde-3-phosphatases were differentially abundant, with one (G3PG) declining and the other (GAPC1, chloroplastic by homology) increasing under imbalanced nutrients at 25°C (**Fig. 5**). Taken together, the maintenance of photosynthetic activity but limited growth at an imbalanced N:P ratio indicate an uncoupling between carbon fixation and cell division. Fixed carbon may be increasingly stored as starch or other storage compounds, as noted by Rosset et al. (2017), facilitated by increased abundance of chloroplastic starch synthase (WAXY; **Fig. 5**). By halting mitosis and reducing energy and nutrient demands, protein synthesis to sustain the photosynthetic apparatus may be prioritized during P deficiency (Li et al. 2016, Rosset et al. 2017).

In agreement with severely impaired cell division, there were corresponding decreases in the abundance of a number of proteins involved in protein translation and cellular growth (**Fig. 5**). Twelve ribosomal proteins were less abundant under the imbalanced regime, with interesting implications for gene expression in dinoflagellates due to a number of unusual gene regulation features (Beauchemin et al. 2012). Gene expression in dinoflagellates appears to be under greater control at the level of translation from mRNA to protein than in many eukaryotes (Roy et al. 2018), a conclusion supported by transcriptome analyses of Symbiodiniaceae under thermal stress (Barshis et al. 2014, Gierz et al. 2017). These results, together with the increase in alkaline phosphatase activity (**Fig. 1H**), reflect cellular acclimation to P limitation and emphasize the relevance of protein-based gene expression assays.

Nutrient imbalance and elevated temperature did not appear to have synergistic effects when observing physiological measures (**Fig. 1**), and there was a strong overlap of nutrient DAPs at each temperature (**Fig. 3C**). While ‘photosynthesis’ was significantly enriched among DAPs due to both temperature and imbalanced nutrient stresses, these DAPs were overwhelmingly less abundant in response to high temperature but more abundant in response to imbalanced N:P (**Figs. 4**, **5**). Whether these responses are antagonistic and result in amplified cellular stress, particularly if thermal stress is more extreme or sustained, is unclear. ‘Response to oxidative stress’ or ‘cell redox homeostasis’ GO terms were enriched among nutrient DAPs at either temperature, indicating that an imbalanced N:P ratio results in oxidative stress independent of temperature (**Table 3**, **4**). Thermal stress is thought to be in part due to increased production of reactive oxygen species (Smith et al. 2005). This discrepancy between stress responses may be due to the thermal stress here being insufficiently severe or prolonged to induce pronounced oxidative stress in addition to the detected protein-folding stresses.

We note that the ‘membrane’ GO term was significant among nutrient DAPs at both temperatures (**Table 3**), and membranes are common targets of oxidative damage (Schieber and Chandel 2014). Limiting phosphorus may impair membrane synthesis and protein repair, as well as reduce the availability of phosphate for ATP regeneration. These inhibited processes may be greatly disruptive to the cell independent of thermal stress and limit the cell’s thermal resilience and capacity for repair. Thirty-seven and 41 ATP-binding proteins from a variety of biological processes were differentially abundant at 25 and 34°C, respectively, as a result of the imbalanced regime (**Tables 3**, **4**). It is unclear whether higher temperatures would have resulted in an enhanced response in combination with imbalanced N:P, but our results suggest that algal symbionts under imbalanced N:P are already experiencing oxidative stress.

### Imbalanced nutrient stresses and coral bleaching

Highly imbalanced N:P ratios as a result of anthropogenic nutrient inputs have been proposed to increase the susceptibility of reef-building corals to thermal bleaching (Wiedenmann et al. 2013, Morris et al. 2019). Here, in culture, imbalanced but ecologically relevant N:P ratios had a larger effect on the *S. microadriaticum* proteome than elevated temperature and reduced population growth to nearly zero. Together with an increase in alkaline phosphatase activity and a near-uniform reduction in ribosomes and other translational components, we conclude that the algal cells were phosphorus-limited under these imbalanced nutrient conditions. Photosynthetic rates were unaffected by nutrient availability, although protein components of light-harvesting complexes and downstream carbon-fixation proteins were more abundant at imbalanced N:P. The hypothesized amplification of, or sensitivity to, thermal stress of *S. microadriaticum* under imbalanced N:P stress was not readily observed in the physiological responses (**Fig. 1**) or at the individual protein level (**Fig. 3**), in contrast to other studies (Wiedenmann et al. 2013, Rosset et al. 2017). This may be due to an insufficient duration or intensity of each stressor in our study. There was, nevertheless, evidence that imbalanced nutrients resulted in increased oxidative stress at 34°C (**Table 4**).

These results have concerning implications for corals on reefs exposed to chronically high N:P, either independent of or in conjunction with thermal stress. Despite the lack of strong direct interactions between thermal stress and phosphorus limitation on algal physiology, the inhibition of algal cell growth under phosphorus limitation may threaten the stability of the cnidarian–dinoflagellate symbiosis (Morris et al. 2019). Severely imbalanced N:P ratios may be sufficient to induce nonthermal bleaching either by directly impairing symbiont cell division or by creating competition for nutrients between the host and symbiont (Pogoreutz et al. 2017). Low rates of symbiont mitosis resulting from phosphorus limitation result in lower symbiont cell densities, as noted by Rosset et al. (2017), who measured a 60% reduction in *Euphyllia* symbiont density under phosphorus limitation compared to nutrient-replete conditions. Low symbiont densities may be a result of a ‘ratchet’ of slow cell division rates, as well as attrition from steady-state expulsion or consumption of symbiont cells by the host (Morris et al. 2019). Such corals with low symbiont densities at normal temperatures would be particularly susceptible to symbiont loss, rapid bleaching and host starvation during high sea surface temperature events. If symbionts are experiencing nutrient-induced oxidative stress prior to any thermal stress, they may be especially at risk.

Experimentation on Symbiodiniaceae cultures has limitations, as symbiont physiology is strongly affected by symbiosis (Bellantuono et al. 2019, Maruyama and Weis 2021). However, it is not currently possible to accurately determine or regulate the experienced nutrient milieu of symbionts *in hospite*, as N and P fluxes between the host and symbiont may be highly dynamic and take many chemical forms, organic or inorganic. Culture experiments allow for informed experimental design and controlled nutrient conditions. Established Symbiodiniaceae cultures also have distinct microbiomes, which may positively or negatively affect algal nutrient transport (Camp et al. 2020, Wuerz et al. 2022). The *S. microadriaticum* culture used here is not axenic, and therefore, the observed responses may be in part a result of bacterial activity, particularly the uptake or release of N or P compounds or other metabolites. In order to estimate the abundance of the bacterial community and, by extension, its contribution to rates of respiration and nutrient uptake, we performed a second search of the mass spectra as before against a sequence database composed of all reviewed bacterial sequences in UniProt (SwissProt). This identified 130 bacterial proteins (**Supplementary Material S8**), with a total protein abundance (as estimated by taking the sum of the intensities of all identified proteins) of approximately 1.5% that of the *S. microadriaticum* proteins (**Supplementary Material S9**). This is consistent with a detectable but small bacterial population, and so we consider bacterial contributions to oxygen and nutrient flux in this study to be minor.

The cnidarian–dinoflagellate symbiosis is based on nutrient exchange, and so the host’s ability to acquire and provide inorganic N and P determines the concentration of nutrients available to the symbionts (Tremblay et al. 2012). Changes in host heterotrophy could therefore serve to buffer or moderate symbiont nutrient stresses (Grottoli et al. 2006, Martinez et al. 2022). The supply of nutrients from host heterotrophy is host-specific (Hughes and Grottoli 2013, Conti-Jerpe et al. 2020) and itself dependent on seawater nutrient concentrations, which, if limited, can impair host feeding and induce starvation (Ezzat et al. 2019). Despite many of the unique features of dinoflagellate biology, the proteins and processes discussed here are universal among photoautotrophic eukaryotes, and so similar effects might be expected in autotrophs or other organisms unable to acquire sufficient phosphorus (e.g. by heterotrophy) (Lapointe 1997). The tight nutrient recycling and regulation of the cnidarian–dinoflagellate symbiosis may make it particularly susceptible to nutrient perturbation. The availability of N and P, which can be greatly impacted by anthropogenic inputs and is therefore subject to management decisions, may contribute to coral resilience due to many factors, including host/symbiont compatibility, disease and thermal extremes (Oakley and Davy 2018, Rosset et al. 2021).

Photosynthesis and nutrient acquisition are central to the function of the cnidarian–dinoflagellate symbiosis and, therefore, the health of coral reefs. We have cataloged the proteomic response of the coral symbiont *S. microadriaticum* to thermal stress and varying nutrient ratios, either of which may cause impaired coral health and bleaching. Thermal effects on the *S. microadriaticum* proteome were consistent with prior physiological or transcript-based studies, principally affecting photosynthesis and protein-folding mechanisms, but the resolution provided by LC–MS/MS allows for a better understanding of these processes and identifying which proteins are responsible. Whether these thermal responses are consistent in nature or intensity across the Symbiodiniaceae is unknown, however. Identifying proteins or proteome responses that are unique to certain species, or a putative ‘core thermal response’ conserved across the Symbiodiniaceae, would greatly enhance our understanding of the cellular processes behind the observed differences in thermal resilience between species. Sufficient coverage and precision of symbiont protein expression may identify genes of relevance for selecting resistant strains, genetic modification or other interventions (Rosset et al. 2021). High absolute concentrations of N and P near the Redfield ratio had little effect on the proteome. The distinct proteome and pathways affected by imbalanced N:P ratios, however, detail a state of greatly impaired symbiont cell division that, prior to any thermal stress, may result in low symbiont densities and greater host bleaching susceptibility or reduced resilience. Nutrient stress resulted in increases in redox homeostasis proteins, suggesting greater susceptibility to redox pressure from the two stressors in combination. By identifying common cellular responses or genes that ameliorate stress from both high temperatures and nutrient limitation, we may greatly increase our understanding of the cell biology of coral bleaching while also improving our ability to intervene.

## Materials and Methods

### Culture conditions

*Symbiodinium microadriaticum* (LaJeunesse 2017) cells were cultured in f/2 medium at 25°C and 40 µmol quanta m^−2^·s^−1^ under a 12 h:12 h, light:dark photoperiod prior to the experiment. Cultures were resuspended in an artificial seawater medium (Coral Pro Salt; Red Sea Aquatics Ltd., Houston, TX, USA) prepared with distilled water and supplemented with inorganic N and P, as follows. After 2 weeks of acclimation to these nutrient concentrations, 5 million cells in 2 mL of medium were transferred to glass culture tubes (*n* = 32 per nutrient condition), then brought to a total volume of 8 mL of medium, with 2 mL gas volume, and maintained at 60 µmol quanta m^−2^·s^−1^. Cultures were acclimated to these light levels for 1 week. Half of the medium was exchanged each day throughout the acclimation and experimental periods to maintain the given N and P concentrations, taking care to avoid removing algal cells. For the high-temperature treatment, the temperature of half of the aliquots (*n* = 16 per nutrient condition) was increased from 25 to 34°C over 1 week and maintained at 34°C for 48 h, while the rest remained at 25°C. Respirometry was conducted on 3–4 aliquots per nutrient × temperature condition, the remaining culture aliquots pelleted by centrifugation, the medium decanted and the cell pellet rapidly frozen at −80°C for subsequent analysis. *Symbiodinium microadriaticum* genetic identity was confirmed by ITS2 sequencing (Macrogen, South Korea).

### Inorganic N and P concentrations

Artificial seawater was supplemented with sodium nitrate and disodium hydrogen phosphate to establish three nutrient conditions: ‘low’ (low N and low P), ‘imbalanced’ (high N and low P) and ‘high’ (high N and high P; **Table 5**). The term ‘low’ is used as a relative descriptor, and we acknowledge that it may not represent limiting N or P in *Symbiodinium*. To determine whether cultures significantly affected the inorganic N and P concentrations by assimilation, the dissolved inorganic nitrogen and dissolved inorganic phosphorus concentrations of both the prepared stock media and culture media at the end of the experiment were analyzed by the NIWA Water Quality Laboratory (*n* = 2 per treatment; Hamilton, New Zealand). As half of the medium was replaced each day, the effective nutrient concentrations experienced by the algae throughout the experiment were assumed to be intermediate between the two measurements (**Table 5**). These concentrations are within the range of values observed on coral reefs worldwide (Szmant 2002, Lapointe et al. 2019, Mies et al. 2020). The high and imbalanced regimes contained a similar concentration of phosphorus, but different concentrations of nitrogen, to directly compare the influence of the relative availability of nitrogen to phosphorus on *S. microadriaticum* ecophysiology.

**Table 5:** The concentrations of dissolved inorganic nitrogen (DIN) and dissolved inorganic phosphorus (DIP) of the initial stock seawater media of the *S. microadriaticum* culture flasks 24 h after exchanging half of the medium on the final day of the experiment and the mean of each

	DIN (µM)			DIP (µM)			N:P		
Condition	Initial Stock	Medium after 24 h	Mean^a^	Initial Stock	Medium after 24 h	Mean	Initial Stock	Medium after 24 h	Mean
Low	2.6	1.0	1.8	0.1	0.3	0.2	27	4	15.5
Imbalanced	26.5	26	26.3	0.4	0.6	0.5	75	42	59
High	4.6	1.3	3.0	0.5	0.6	0.6	9	2	5.5

a 50% of the culture medium was exchanged with new stock medium daily.

### Physiological measurements

Several physiological parameters were measured to inform our interpretation of the proteome data. The minimum (*F*_0_), maximum (*F*_m_ or *F*_m_′) and steady-state (*F*_t_) fluorescence of chlorophyll *a* were measured mid-photoperiod by pulse-amplitude modulated fluorometry (Diving PAM; Walz, Germany) in situ through the culture vessel wall using the fiberoptic probe. The PAM fluorometer settings are as follows: measuring intensity = 8, saturation intensity = 8, saturation width = 0.8 s and dampening = 2. The maximum quantum efficiency of PSII was calculated as (*F*_m_ − *F*_0_)/*F*_m_ = *F*_v_/*F*_m_ from measurements after 20 min of dark adaptation. The effective quantum efficiency of PSII was calculated as (*F*_m_′ − *F*_t_)/*F*_m_′ = Δ*F*/*F*_m_′. Photosynthetic and respiratory oxygen fluxes of culture aliquots were measured (*n* = 3–4 per treatment) at the appropriate experimental temperature for 30 min each in the light (80 µmol quanta m^−2^·s^−1^ from an incandescent lamp) and dark using a 1.8-mL chamber, stir bar and optic oxygen sensor (Fibox 3; Presens GmbH, Regensburg, Germany). These culture aliquots were then pelleted by centrifugation, the supernatant removed and the photosynthetic pigments extracted in 70% ethanol for >1 week at −20°C. The cell fraction was retained for cell counts by fluorescence microscopy (below), and the chlorophyll extract was adjusted to 95% ethanol and pigments quantified by absorbance using the chl *a* and total carotenoid functions of Lichtenthaler and Buschmann (2001). Culture cell population growth was assessed by cell counts of subsamples from the first and final days of the experimental period. Cell autofluorescence micrographs (*n* = 12 per sample) were captured by fluorescence microscopy (IN Cell 6500 HS; GE Healthcare, Sydney, Australia, ex. 642 nm), and cells were quantified using IN Carta software (GE Life Sciences). To assess whether algal cells were experiencing phosphorus limitation, we measured the activity of alkaline phosphatase, which is involved in phosphorus acquisition and homeostasis of snap-frozen samples (*n* = 4) using a methylumbelliferyl fluorometric assay (Kelly et al. 2019).

Physiological datasets were assessed by two-way analysis of variance (ANOVA) to compare the effects of temperature and nutrient regime (α = 0.05). If ANOVA assumptions were not met after transformation, a Kruskal–Wallis test was performed (α = 0.05). In addition, the effect of temperature within the nutrient regime was also tested using an unpaired two-tailed *t*-test or a Mann–Whitney *U*-test if assumptions of normality were not met (α = 0.05). For PSII quantum efficiency data, unpaired two-tailed *t*-tests not assuming constant variance were used to compare measurements of control and treatment cultures daily (α = 0.01). For the PSII quantum efficiency measurements of cultures under the imbalanced nutrient regime, a repeated measures ANOVA was performed. All *P*-values were Bonferroni-adjusted, and data points beyond 2 SD of the mean were removed as outliers. All tests were performed using GraphPad Prism 7.04 (GraphPad Software, California) and SPSS 25 (IBM, New York).

### Protein extraction

Proteins were extracted, solubilized and digested using a filter-aided sample preparation method modified from Wiśniewski et al., (2009). Frozen algal samples were washed twice with water, suspended in 5% sodium deoxycholate buffer and lysed with a probe ultrasonicator (20 × 2 s pulses) on ice. β-mercaptoethanol was added to 1% final volume, and the sample incubated at 85°C for 20 min to denature proteins. Two volumes of ethyl acetate were added, and the sample was vortexed for 1 min and then centrifuged for 1 min at 10,000×*g*. The upper ethyl acetate layer was discarded. This wash was repeated three times to remove photosynthetic pigments, and then the lower aqueous layer containing dissolved proteins was transferred to a molecular weight cutoff filter (Amicon Ultra 30 kDa 0.5 mL; Merck Millipore, Auckland, New Zealand), leaving pelleted debris behind. Proteins were concentrated to 20 µL by centrifugation (15 min 14,000×*g*) and washed twice with 380 µL 50 mM Tris buffer (pH 8.2), before being resuspended in 400 µL of 50 mM Tris buffer. A 10 µL subsample was acidified and pelleted to remove deoxycholate, and the total protein was quantified by protein-binding dye fluorescence (Qubit 2.0; ThermoFisher Scientific, USA). Trypsin was added (1:100 enzyme:protein mass), and the protein digested for 18 h at 37°C. The tryptic peptides were collected through the 30 kDa filter by centrifugation, and formic acid was added to 1% final volume to terminate digestion and precipitate the deoxycholate. The precipitate was pelleted by centrifugation for 1 min at 16,000×*g*, and the supernatant, containing peptides, was transferred to a new tube. The peptides were then desalted with C18 tips (Omix Bond Elut; Agilent Technologies, Santa Clara, CA, USA).

### Chromatography and mass spectrometry

Samples were analyzed by LC–MS/MS using methods similar to those of Oakley et al. (2017), with a nonlinear 300 min gradient (buffer A: 0.1% formic acid; buffer B: 80% acetonitrile, 0.1% formic acid) at 0.3 μL min^–1^ on an Acclaim PepMap C18, 150 cm long, 75 μm inner diameter, 3 μm particle size, 100 Å column (#160321, ThermoFisher Scientific) and Ultimate 3000 liquid chromatograph (ThermoFisher Scientific). Peptides were analyzed with an LTQ Orbitrap XL (ThermoFisher Scientific) by injection at a 2.2 kV spray voltage and a resolution of 30,000. The top six MS peaks were analyzed by the ion trap, rejecting +1 charge states with dynamic exclusion enabled (180 s). The instruments were operated with Chromeleon Xpress (v2.11.0.2914), Xcalibur (v2.1) and TunePlus (v2.5.5) (ThermoFisher Scientific). Each sample was analyzed twice as technical replicates.

Protein identification was conducted using the Andromeda search engine in MaxQuant (1.6.3.4) against the *S. microadriaticum* trEMBL database (UniProt taxon 2951), plus common contaminants (Cox and Mann 2008, Cox et al. 2014). False discovery rate (FDR) thresholds were set at 1% for peptide and protein search matches, and a minimum of two peptides per protein were required for identification. Searches assumed trypsin digestion with a maximum of two missed cleavages. Oxidation of methionine and acetylation of the protein N-terminus were specified as variable modifications, and carbamidomethylation of cysteine was specified as a fixed modification. Sequence matches were grouped into protein groups by parsimony and quantified by label-free quantification intensity with the ‘match between runs’ feature enabled (Cox et al. 2014).

### Protein abundance analysis

Known false matches and contaminant proteins were removed from the dataset, and all protein label-free quantification intensity values were log_2_-transformed. The resulting data were analyzed for significance between treatments by polyStest (Schwämmle et al. 2020). Significant proteins were determined by the polyStest algorithm as those with an FDR ≤ 5% and a log_2_ FC whose absolute value was >0.25. Principal component analyses of the log_2_-transformed protein abundance data were conducted by singular value decomposition with imputation and unit variance scaling using the ClustVis package (Metsalu and Vilo 2015). Venn diagrams were generated using jvenn (Bardou et al., 2014) and eulerAPE (v. 3.0.0, Micallef & Rodgers, 2014).

### Protein functional enrichment analysis

Identified proteins were annotated with GO terms by sequence homology using PANNZER2 (Törönen et al. 2018). GO term enrichment analysis of treatment comparisons was performed using the package topGO (v. 2.38.1, Alexa et al. 2006) in R (v. 4.0 (R Core Team 2021). Using all proteins identified in the dataset as the ‘background’, GO terms of significant (FDR < 5%) proteins between treatments were tested for enrichment (*P* < 0.1) in all GO domains using Fisher’s exact test with a node size of 2.

## Supplementary Material

pcac175_Supp

## Data Availability

The mass spectrometry proteomics data have been deposited to the ProteomeXchange Consortium (http://proteomecentral.proteomexchange.org) via the PRIDE partner repository (Perez-Riverol et al. 2019) with the dataset identifier PXD022821.
