# Supplementary material for: The Symbiodinium Proteome Response to Thermal and Nutrient Stresses
Source: Plant Cell Physiol. 2022 Dec 24;64(4):433–47. doi: 10.1093/pcp/pcac175 (PMC10109209; doi:10.1093/pcp/pcac175)
Supplement: pcac175_Supp [file pcac175_supp.zip › suppl_data/pcp-2022-e-00267-File015.pdf]

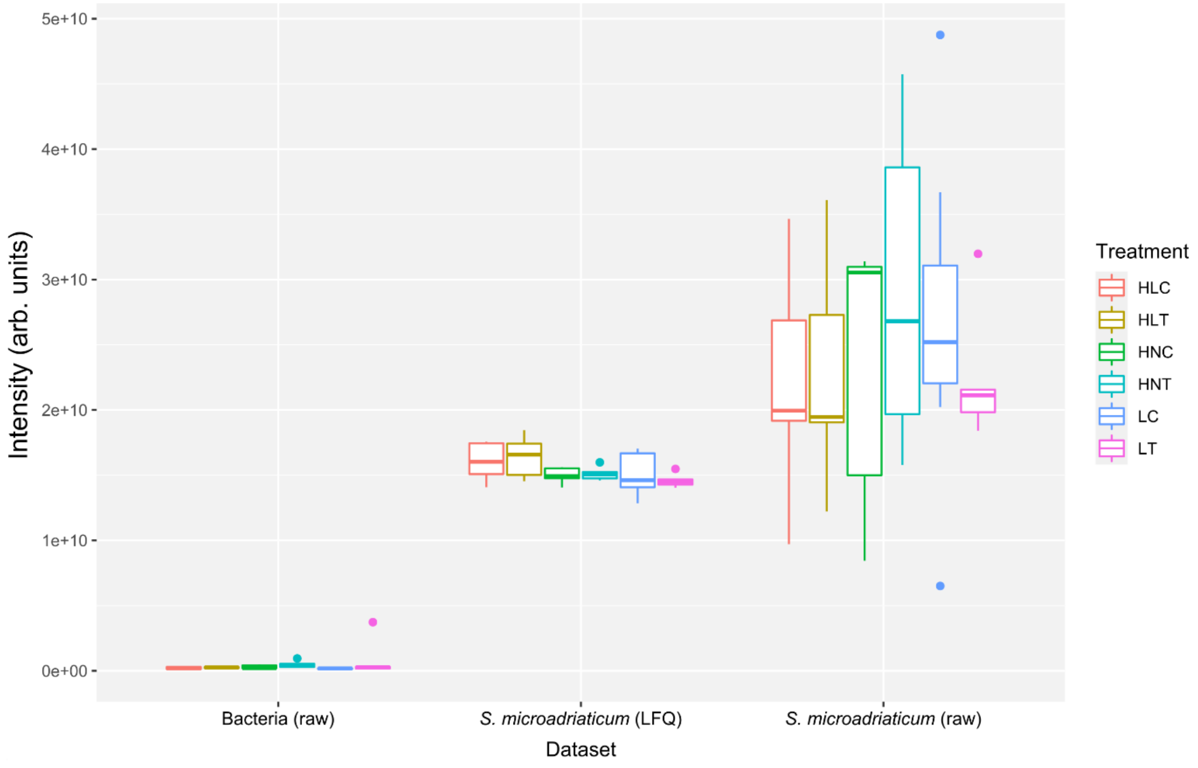

Supplementary Material S9 Total summed intensities of *Symbiodinium* and bacterial proteins in each sample. Bacteria values refer to mass spectra searched against all bacteria sequences in SwissProt. "Raw" refers to summed protein intensities without label-free quantification (LFQ) normalisation. Treatments: LC = low nutrient, control temperature; LT = low nutrient, high temperature; HNC = high nutrient, control temperature; HNT = high nutrient, high temperature; HLC = imbalanced nutrient, control temperature; HLT = imbalanced nutrient, high temperature.
